# Comparison of data mining and allometric model in estimation of tree biomass

**DOI:** 10.1186/s12859-015-0662-5

**Published:** 2015-08-07

**Authors:** Carlos R. Sanquetta, Jaime Wojciechowski, Ana P. Dalla Corte, Alexandre Behling, Sylvio Péllico Netto, Aurélio L. Rodrigues, Mateus N. I. Sanquetta

**Affiliations:** Forest Science Department, Federal University of Paraná, 900 Lothário Meissner Avenue, Curitiba, Paraná Brazil

**Keywords:** Accuracy, Carbon, Forests, Instance, Modeling

## Abstract

**Background:**

The traditional method used to estimate tree biomass is allometry. In this method, models are tested and equations fitted by regression usually applying ordinary least squares, though other analogous methods are also used for this purpose. Due to the nature of tree biomass data, the assumptions of regression are not always accomplished, bringing uncertainties to the inferences. This article demonstrates that the Data Mining (DM) technique can be used as an alternative to traditional regression approach to estimate tree biomass in the Atlantic Forest, providing better results than allometry, and demonstrating simplicity, versatility and flexibility to apply to a wide range of conditions.

**Results:**

Various DM approaches were examined regarding distance, number of neighbors and weighting, by using 180 trees coming from environmental restoration plantations in the Atlantic Forest biome. The best results were attained using the Chebishev distance, *1/d* weighting and 5 neighbors. Increasing number of neighbors did not improve estimates. We also analyze the effect of the size of data set and number of variables in the results. The complete data set and the maximum number of predicting variables provided the best fitting. We compare DM to Schumacher-Hall model and the results showed a gain of up to 16.5 % in reduction of the standard error of estimate.

**Conclusion:**

It was concluded that Data Mining can provide accurate estimates of tree biomass and can be successfully used for this purpose in environmental restoration plantations in the Atlantic Forest. This technique provides lower standard error of estimate than the Schumacher-Hall model and has the advantage of not requiring some statistical assumptions as do the regression models. Flexibility, versatility and simplicity are attributes of DM that corroborates its great potential for similar applications.

## Background

Tropical forests are considered important sinks of global carbon [1], storing about 60 % of the living biomass (298 billion tons of carbon). In the last 10 years, 90,000 km^2^ of tropical forests were decimated, representing 70 % of overall loss of forests [2]. Based on current rates of forest fragmentation, the loss of carbon stocks can produce more than 150 million tons of carbon emissions to the atmosphere annually, with losses that go beyond the anthropic destruction of forests [3, 4]. Deforestation is considered a key factor of global climate changes [5], as the decline of biomass in forest patches could be a significant source of greenhouse gases released after cutting and burning of vegetation [1, 3, 6].

Biomass is a crucial variable for the quantification of stock and dynamics of carbon in forests. Most of the biomass that forests store is concentrated in the tree component of the community [7]. Bole, branches, foliage and roots comprise the major fraction of this biomass, although dead wood, litter and organic carbon in the soil are also important reservoirs for the carbon cycle in forests [8, 9].

Despite of the importance of biomass in the quantification of carbon in trees, their direct determination is complex, costly and destructive. For this reason, usually it is done in an indirect way by the use of allometric models via regression or by application of expansion factors [9–12].

Expansion factors are simpler of building up and to apply, but allometric models are more recommended due to the increased flexibility to describe variations in tree architecture and in compartmentalization of biomass [12, 13].

A crucial aspect in quantification of biomass of individual trees is the large natural variability in data, particularly for native species of tropical and subtropical regions. A single mathematical formulation may not be able to reproduce such a huge natural variation. This factor affects the quality of the model fitting and may provide spurious estimates. Another feature of the allometric models is that when using the regression technique some assumptions must be accomplished. These assumptions are the following: additivity and linearity, independence of residuals, homoscedasticity, and normality of residuals [14].

The Data Mining technique (DM) has the purpose of searching useful information from data sets [15, 16]. This technique, used in learning algorithms, whose metrics can be found in Albert [17] and Bradzil [18], is already widespread in several areas and applications. However, its potential has been little explored to estimate biomass stocks in forests. Recently, Sanquetta et al. [19] introduced its use to estimate tree carbon stock, concluding that it can produce estimates that are as reliable as those obtained by the traditional methods used for this purpose, such as allometry.

This study explores the DM technique as an alternative to allometric models used in tree biomass estimation. The technique is demonstrated for modeling total dry biomass (aboveground + belowground) from data collected in 180 individual trees belonging to various native species planted for environmental restoration purpose in the Atlantic Forest biome, Brazil.

## Results

### Original untransformed 180 data series

**Fig. 1:**
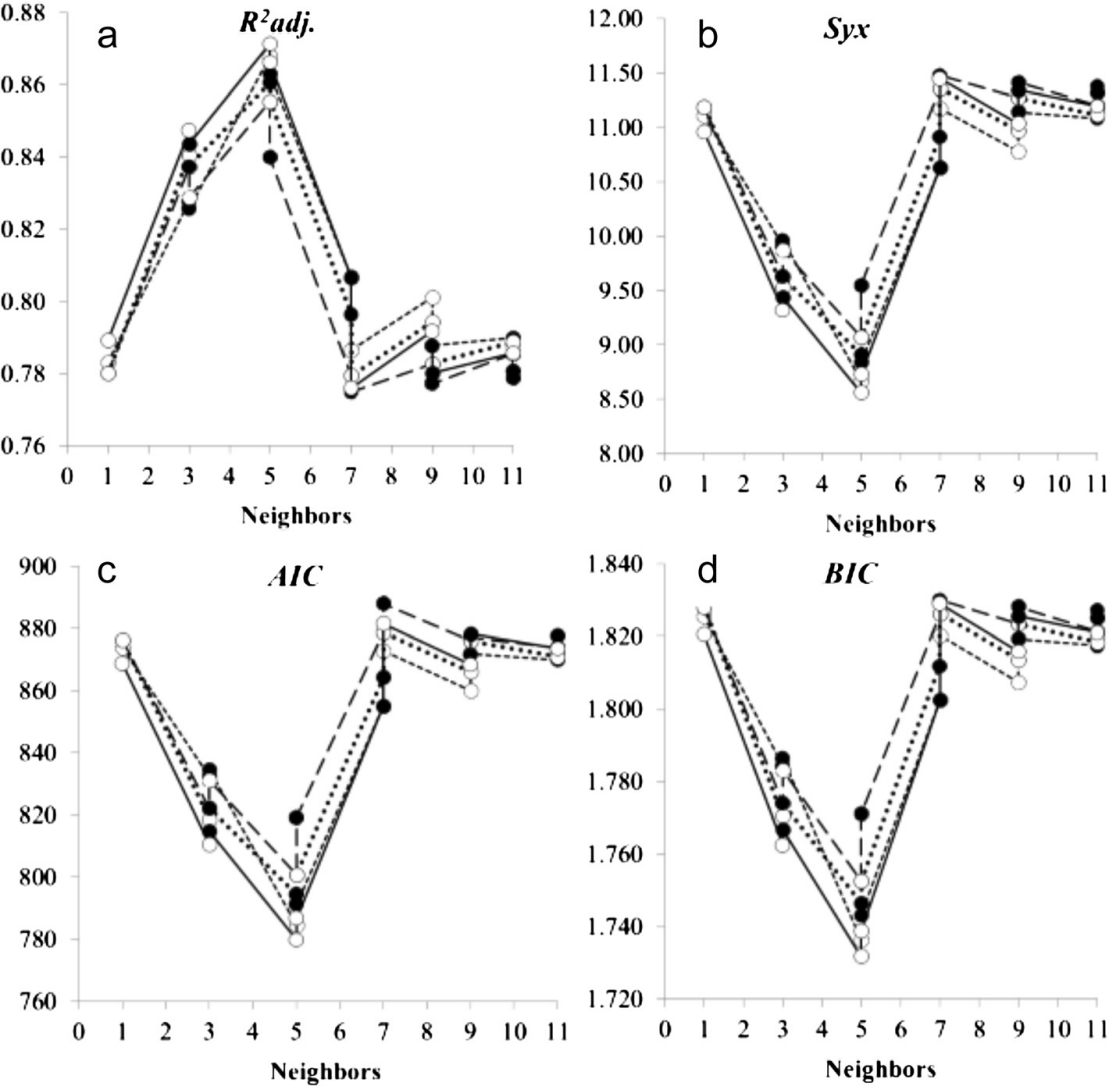
Statistical criteria of model selection applied to 180 data of individual biomass of native trees of the Atlantic Forest, using Data Mining. (Chebyshev distance, Manhattan distance, Quadratic Euclidean distance, Euclidean distance; ●:1/*d* ^2^, ○: 1/*d*; all variables)

Biomass modeled with the original data set (180 untransformed values) using the Chebyshev distance gave the best goodness of fit as measured by *R* ^*2*^ *adj.*, *Syx*, *AIC* and *BIC*, as well as by the residual analysis. The use of the full set of independent variables, i.e., *dbh*, *dm*, *ht*, *hc*, *da*, and *db*, provided the most accurate biomass estimates, when compared to the reduced number of predictor variables. The worst result was obtained by using exclusively *dbh* as the independent variable. Differences between *1/d* and *1/d* ^*2*^ weighting were also noticed, and, in general, the former was found to have better performance as compared to the latter. The most accurate biomass estimation was obtained with 5 neighbors. The analysis of neighborhood indicated that biomass estimation accuracy increased from 1 to 5 neighbors, but decreased afterwards (Fig. 1). It suggests that there is an optimum number of neighbors in DM modeling of biomass and that a few number of neighbors might cause noise, but conversely a greater number would lead to losing accuracy.

**Fig. 2:**
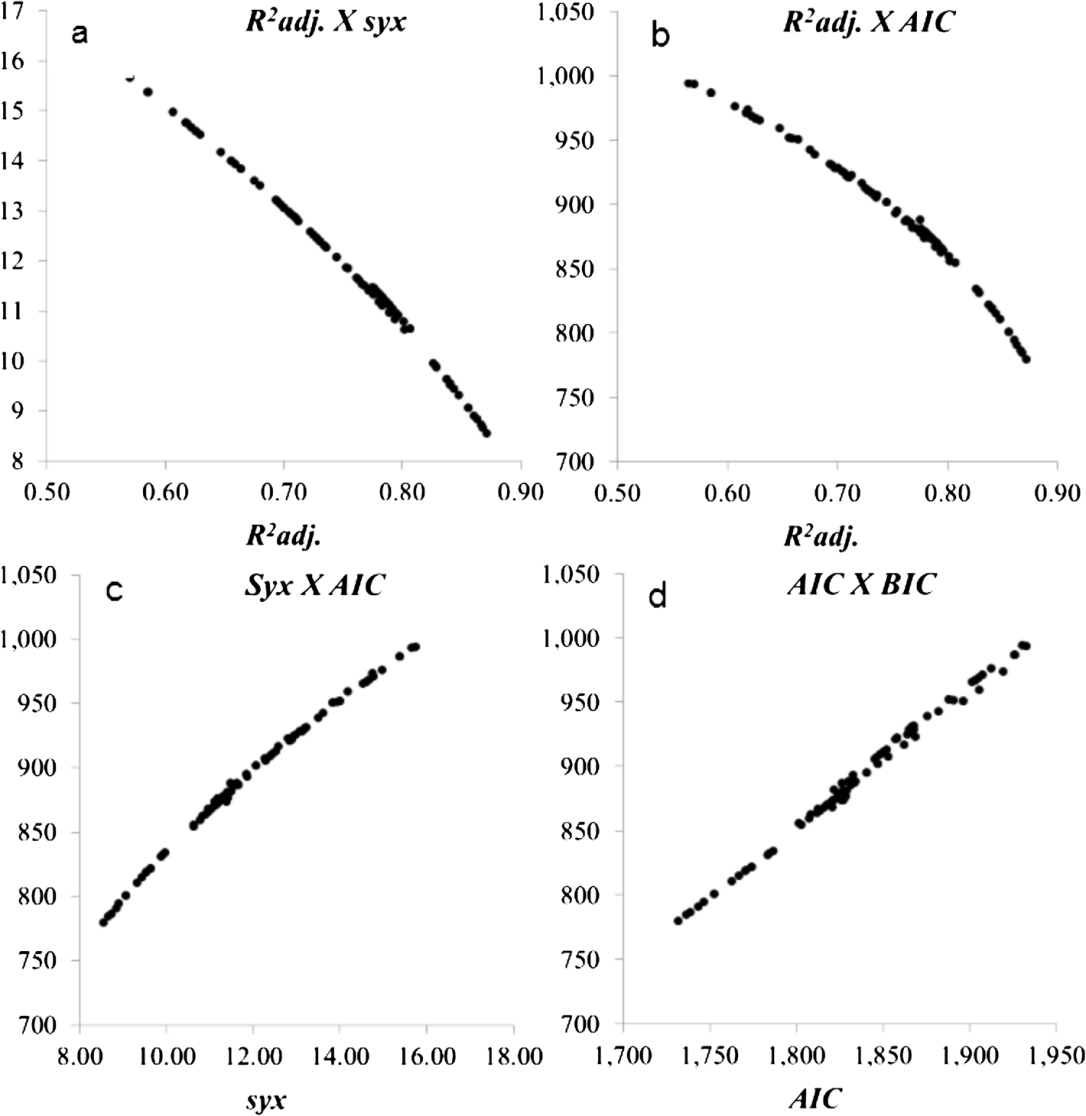
Relationship between statistical criteria for model selection applied to the data of individual biomass of native trees of Atlantic Forest, Brazil (R² adj: adjusted coefficient of determination; syx: standard error of estimate; AIC: Akaike Information Criterion; BIC: Bayesian Information Criterion)

A strong relationship was found among the selection criteria of models. *R* ^*2*^ *adj.* x *Syx*, *R* ^*2*^ *adj.* x *AIC* relations were of negative slightly curvilinear type. On the other hand, *Syx* x *AIC* relations was positive curvilinear. *AIC* x *BIC* association was positive and straight (Fig. 2). This indicates that all the criteria for selecting models lead to the same results.

### Logarithmic transformation and reduced series of data

**Fig. 3:**
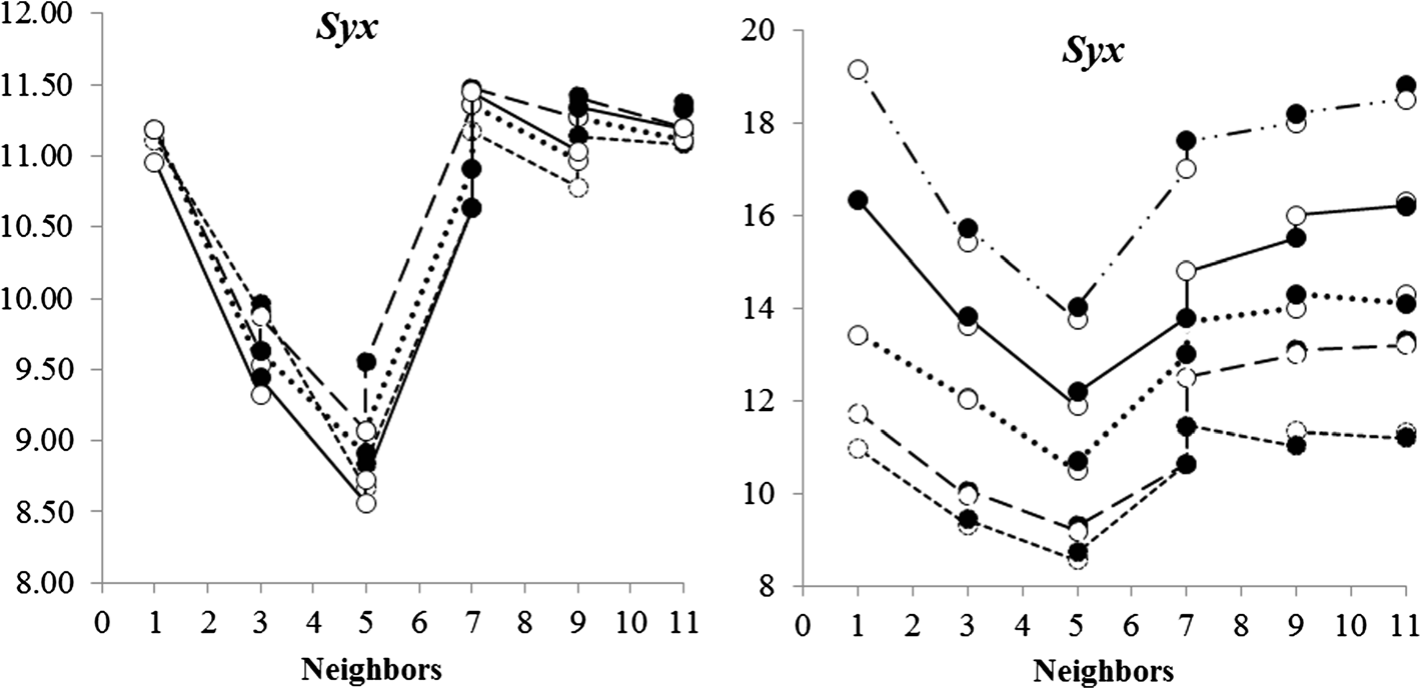
Statistical criteria of model selection applied to the data of individual biomass of native trees of the Atlantic Forest, using Data Mining: **a** - log transformed data; **b** - reduced data size. (Chebyshev distance, Manhattan distance, Quadratic Euclidean distance, Euclidean distance; ●:1/*d* ^2^, ○: 1/*d*; all variables)

The logarithmic transformation of the independent and dependent variables do not lead to improvement in the estimates. The values of *Syx* for estimates with the transformed variables remained close to the corresponding ones without transformation (Fig. 3a); the same happened with the other criteria. It indicates that logarithm transformation in DM applications do not improve model accuracy as it happens when using regression models for tree biomass estimation purposes.

The effect of reducing the number of data in the application of DM technique was evidenced. Results were much better with the use of the full range of data (180 values) and worst with the most reduced series (50 values) (Fig. 3b). Reduction in the number of data has not changed the results of preliminary analyses, i.e., the best results are still those obtained with 5 neighbors and with the full set of variables in relation to the use of *dbh* only. Thus, it is shown that the best performance of DM technique is given with the maximum number of data and independent variables.

### Variation in distances and comparison with the Schumacher-Hall’s classic allometric model

The Euclidean distance is the most commonly used in DM application by Classification Based on Instances for its simplicity and recognition as appropriate under various circumstances. Our findings have showed that the Chebyshev Distance provided better results when compared to the Euclidean distance and the others evaluated (Fig. 3a).

The Schumacher-Hall equation adjusted to the complete series of data provided satisfactory results, with selection criteria for models comparable to those obtained with application of DM (Table 1). The resulting equation was the following:

**Table 1:** Criteria for model selection for the Schumacher-Hall equation applied to 180 data of individual biomass of native species of the Atlantic Forest biome, Brazil

R^2^adj.	Syx	AIC	BIC
0.8082	10.4521	847.83	1792.14

1 $$ \ln (w)=-1.390796+1.051491\;\left[ \ln (dbh)\right]+1.084280\;\left[ \ln (ht)\right]+{e}_i $$

Comparative analysis of tree biomass estimates obtained with the selected DM model and the Schumacher-Hall equation indicated that there was no statistical difference between them. However, when comparing the Data Mining models to Schumacher-Hall equation it was evidenced an expressive gain in terms of accuracy in tree biomass estimation. All DM models showed smaller *Syx* values: 8.6 % for Quadratic Euclidean Distance, 13.2 % for Euclidean Distance, 15.4 % for Manhattan Distance and 16.5 % for the Chebishev Distance.

**Fig. 4:**
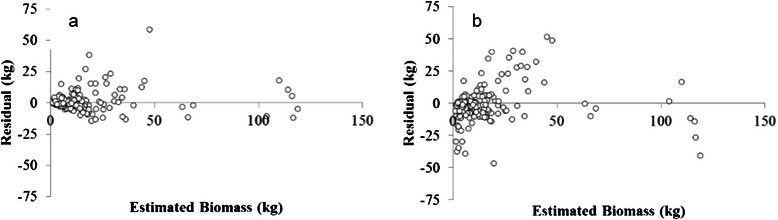
Graphical analysis of residuals of models applied to the data of individual biomass of native trees of the Atlantic Forest: **a** - Data Mining, **b** - Schumacher-Hall model

Furthermore, the graphical analysis of residuals of biomass revealed that the estimates obtained by DM (180 observations, series of data not transformed, all independent variables and Chebyshev distance) presented balanced residuals distribution and lower dispersion along the estimation line when compared to Schumacher-Hall model estimates (Fig. 4).

Therefore, the results of this study demonstrates that DM can improve biomass estimation of individual trees and can be successfully employed for this purpose, reducing uncertainty in carbon stock quantification in forests.

## Discussion

The Atlantic Forest, one of the main hotspots of biodiversity in the Neotropics, lost more than 90 % of its original area in the last 150 years due to anthropic processes [20]. These processes were responsible for the release of millions of tons of greenhouse gases into the atmosphere, contributing greatly to the overall balance of carbon. Recent efforts have presented positive effects in order to contain the deforestation in the Brazil, as well as to restore ecosystems highly disturbed. Restoration plantations are also an important requirement in the new Brazilian Forest Code sanctioned in 2012, aimed at recovering natural ecosystems in the rural areas [21–23].

A key indicator of the success of environmental restoration plantations in the Atlantic Forest is its carbon stock. Estimates of current carbon stocks in this biome depend on the knowledge of their biomass [24]. Biomass quantification is usually performed by extrapolation from the plots of forest inventory, where structural variables of the forest are measured, such as diameter and height of the trees [25] and even the wood density, when possible.

Most forest biomass studies are based upon allometric models due to the difficulty of carrying out direct measurements, which involve cut and weighing of trees. Although the allometry technique is already known and widespread [26], few models are available for estimation of biomass in tropical forests [13, 27]. For the Atlantic Forest there are few equations [28–31] some of which have recently been developed specifically for restoration plantations [24, 29–33].

Allometric models generally are fitted using the log transformation approach followed by linear regression assuming linearity and additivity of data, and homoscedasticity, independence and normality of residuals [14].

The assumptions of regression modeling by applying the Schumacher-Hall equation indicated normality (Shapiro-Wilk test - 0.98; *p* > 0.01), homoscedasticity (White test - 34.90; *p* < 0.01) and independence of residuals (Durbin-Watson test - 1.49; *p* < 0.05). Therefore, the use of ordinary linear regression would not be a trouble. However, for trees, in particular, ideal allometry data are strongly heteroscedastic, exhibiting increasing variation in biomass with increasing diameter [34, 35].

One of the assumptions in the classical linear regression model is that the errors *ê*_*i*_ of the dependent variable in relation to the independent variable in the fitted model presents common variance *σ* ^2^, and this constraint is known as homoscedasticity of errors. When errors do not have constant variance occurs heteroscedasticity. One of the ways to detect it is to construct a graph of estimated residuals *ê*_*i*_ in relation to *ŷ*_*i*_ for different values of *x*_*i*_ and check whether there is any systematic pattern in its distribution, i.e. shows a heterogeneous character [36]. Also, statistical tests can be used to check the heteroscedasticity of errors in the regression model, such as that of White [37], which involves all the explanatory variables, their squares and cross-products.

The importance of evaluating heteroscedasticity is commented by Gujarati and Porter [38]. If this constraint is not met the ordinary least squares estimators (OLS) will present bias and no consistency. These estimators present no longer minimum variance or efficiency, therefore cease to be linear and not biased (BLUE). Such estimators are obtained by the method of weighted least squares, always when heteroscedasticity of variances of the errors is known. In these circumstances the variances of the estimators of least squares are not given by standard formulae of OLS, however if these formulas are used, t and F tests based on them can be misleading, resulting in erroneous conclusions. If heteroscedasticity is detected, it is not a simple task to correct it. For large samples it is possible to make inferences on the basis of the White’s test and, in the case of small samples, using the OLS residuals it is possible to transform the original data, thus eliminating heteroscedasticity. Maddala [36] presents the proof for these two situations.

Nonlinear fitting without log transformation is an alternative to biomass allometry [35]. However, neither linear regression on log-transformed data nor standard nonlinear regression is inherently superior. Which method performs better depends on the distribution of the error. In most cases error is distributed such that log transformed linear regression will produce more accurate parameter and confidence intervals estimates [39].

Methodological options beyond regression should be sought to improve tree biomass estimation. Data Mining represents an option with great potential for estimation of forest biomass in many situations regardless the features of data and the nature of the variables taken into account. The virtues of DM include versatility and flexibility. The features of the data and variables used in modeling are not as restrictive as compared to regression and there is no need to meet its statistical assumptions. In addition, the calculations are fairly simpler when compared to nonlinear regression procedures and its implementation on computer is not complicate. First of all, it proved to be able to give more accurate estimates of tree biomass than the traditional method [19].

## Conclusions

- Data Mining (DM) technique makes it possible to obtain accurate estimates of dry biomass of individual trees in the Atlantic Forest;
- The estimates obtained with DM technique are comparable or even more accurate than those obtained by the traditional allometric method, which in this study is represented by the equation of Schumacher-Hall;
- To generate more accurate estimates, the DM technique needs the greatest possible quantity of data and of independent variables;
- The logarithmic transformation did not improve the estimated results;
- The Chebyshev distance proved to be the most appropriate among the evaluated procedures;
- The main virtues of the DM technique are versatility, flexibility and simplicity, besides not requiring to meet the regression assumptions.

## Methods

### Data

For this study, were used data of total dry biomass (above and belowground) collected from 180 trees of 79 native species from the Atlantic Forest, coming from environmental restoration plantations located in the Seropedica municipality, Rio de Janeiro State, southeastern Brazil. The property belongs to the Barbosa Lima Sobrinho Thermoelectric Plant. The stand plantings’ spacing ranged from 1.5 × 1.5 m; 2.0 × 1.5 m; 2.0 × 2.0 m; 3.0 × 2.0 m and their ages ranged from 2 to 6 years (base 2008).

Before direct biomass determination, the diameter at breast height (*dbh*), mean crown diameter (*dm*), total height (*ht*) and height of the lowest living branch (*hc*) were measured. Determination of dry biomass was preceded by weighting the fresh biomass in the field. The simple separation destructive method was adopted [40]. Wood, branches, leaves and root samples were taken and brought to the laboratory. Dry biomass was obtained by direct relationship with fresh biomass after drying the samples in an oven with air circulation at 70° C until constant weight. Wood discs from the tree base, middle and top of the main bole were taken in order to determine apparent (*da*) and basic wood (*db*) density by using the hydrostatic balance method.

### Estimates

Estimates of biomass were carried out with the DM technique called Instance-Based Classification [15], which consists in the search for information of neighboring elements to obtain an estimate of an element of interest, i.e., values in the data set are searched for the ones closest to the object value of the estimate. The estimate is given by the average of the values of nearest neighbors to the element to be estimated. The technique is based on the premise that instances, when the vectors formed by their dimensions are closer, tend to belong to the same class. This proximity can be measured by the distance between the vectors formed by independent variables related to the object of study. This technique method is similar to the kNN (k Nearest Neighbors) approach used in satellite imagery analysis applied to forestry [41, 42].

**Fig. 5:**
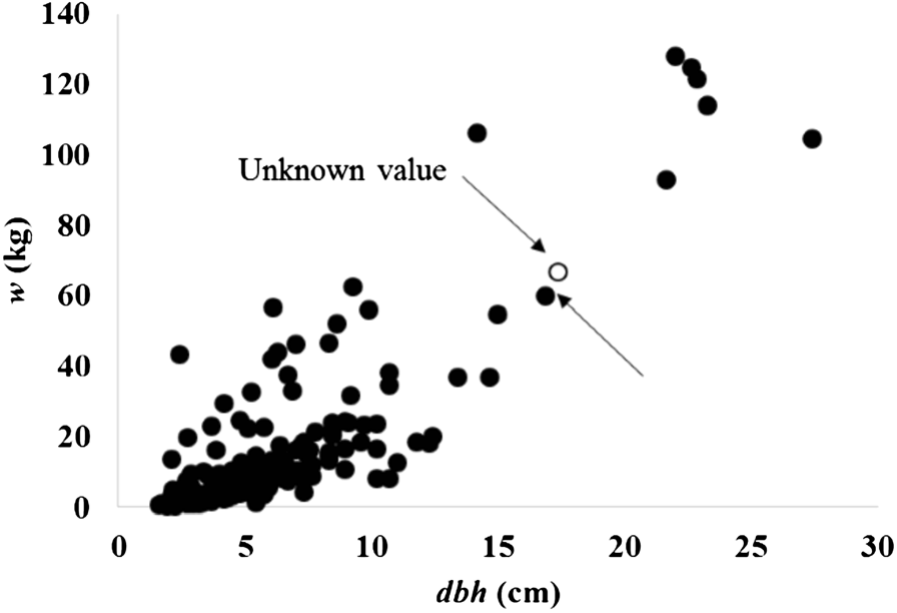
Exemplification of the DM technique through the nearest neighbor distance

Figure 5 illustrates the practical operation of the DM technique. The graph contains the observed values of total dry biomass of individual trees used in this study and their *dbh*. In this illustration, having a tree to estimate the biomass (unknown value shown in Fig. 5), it is possible to calculate the distance between the vector formed by their *dbh* dimensions and biomass of all the trees in the sample. When one finds the tree of shortest distance to the tree of unknown biomass, the estimate of the biomass of that tree will be the biomass of the nearest neighbor(s).

For the comparison of an instance with the others, is used a technique known as cross-validation, in which each instance is compared to the others of the sample, being selected the instance with less distance. It is common in this type of approach the occurrence of “noise”, that are instances not well positioned in the base [16]. This means that, even if a given instance has its dimensions within the pattern of the others, it has the value of the dependent variable very different from values of other instances. This instance, then, is called “noise” (different from an ‘*outlier*’ which is an atypical and not acceptable value for the data set). The problem that could occur, if the value of this instance is considered as a “noise”, then it would be out of the normal patterns and could cause errors in the estimate. To minimize such vulnerability the technique of Classification Based on Instance uses some variations of the method. Thus, one can use the nearest neighbor (more susceptible to noise) or make a weighting with other neighbors to dilute the error. In this case, the information of the nearest neighbors is used, which favors the smaller distance, using the inverse of the distance as weighting procedure. This procedure is indicated in the study of Bradzil [18], which estimates one to five nearest neighbors.

The effect of the number of neighbors on the biomass estimates was examined. The criteria used in this study to define the neighborhood were the following: only one, 3, 5, 7, 9 and 11 nearest neighbors. In addition, the following distances were evaluated to define such neighbors:

- Euclidean Distance:
  2 $$ d\left(p,\kern0.1em q\right)=\sqrt{{\left(X{1}_p-X{1}_q\right)}^2+{\left(X{2}_p-X{2}_q\right)}^2+{\left(X{3}_p-X{3}_q\right)}^2+\dots +{\left(X{n}_p-X{n}_q\right)}^2} $$
- Quadratic Euclidean distance:
  3 $$ d{\left(p,q\right)}^2={\left(X{1}_p-X{1}_q\right)}^2+{\left(X{2}_p-X{2}_q\right)}^2+{\left(X{3}_p-X{3}_q\right)}^2+\dots +{\left(X{n}_p-X{n}_q\right)}^2 $$
- Manhattan Distance:
  4 $$ dm\left(p,q\right)={\left|X{1}_p-X{1}_q\right|}^2+{\left|X{2}_p-X{2}_q\right|}^2+{\left|X{3}_p-X{3}_q\right|}^2+\dots +{\left|X{n}_p-X{n}_q\right|}^2 $$
- Chebyshev Distance:
  5 $$ dc\left(p,q\right)= \max \left(\left|X{1}_p-X{1}_q\right|\right);\left(\left|X{2}_p-X{2}_q\right|\right);\left(\left|X{3}_p-X{3}_q\right|\right);\dots \left(\left|X{n}_p-X{n}_q\right|\right) $$

Where:

*X*_*1*_, *X*_*2*_, *X*_*3*_, …, *X*_*n*_ = independent variables (*dbh*, *dm*, *ht*, *hc*, da, and *db*);

*X*_*np*_ *, X*_*npq*_ = any combination of two values (*p* and *q*) specific to an independent variable;

*n* = number of cases of actual data.

The smaller the distance, the closest is an element of that target estimate. To define the neighbors two choices of weighting were employed: the inverse of the distance [1/ *d(p,q)*] and the inverse of the square of their distance [1/ *d(p,q)* ^2^]. The estimate itself is then obtained by the arithmetic mean of the values of neighboring elements found after the weighting of distances.

To obtain the estimate of the value of biomass of individual trees that have only one or more of the so-called independent variables (in the case *dbh*, *dm*, *ht*, *hc* and *db*), one can calculate the distances by using these variables and searching in the set of real data for the value of actual biomass nearest to it (or the average of the nearest three or five). In this study the following variables were analyzed:

- *dbh*;
- *dbh* and *ht*;
- *dbh*, *ht*, *da*, *db*;
- *dbh*, *dm*, *ht*, *hc*, *da*, *db* (all).

The logarithm transformation (neperian) of the independent and dependent variables were also examined. As in allometry, log transformation generally improve model quality because of the reduction in data dispersion. Therefore, we tested its effect because it could lead to improved estimation when using the DM approach to estimate biomass. Moreover, five variations in the data set were also investigated to analyze the effect of the number of observations on the results: for entire series of data (180) and for the randomly reduced series (150, 100, 70 and 50 data). The objective of this analysis was to verify if a smaller data set could worsen the biomass estimation with DM.

DM technique allows several variations regarding number of neighbors, number and transformation of variables, size of data set, distances, weighting, and so on. Due to this complexity and the inherent principle of cross-validation, in which each observation is compared to all the others in the sample, a software was developed in JAVA platform and 1760 simulations with these variations were performed, greatly reducing the time to make the calculations.

### Comparison with the classical Schumacher-Hall model

A comparison between DM estimates and those coming from a classic allometric model was performed. The Schumacher-Hall model has been used for individual tree volume estimation, but has also application for the biomass calculation, as it is presented in (6):

6 $$ \ln (w)=a+b\left[ \ln (dbh)\right]+c\left[ \ln (ht)\right]+{e}_i $$

Where:

*a, b*, *c* = coefficients to be adjusted.

*ln =* neperian logarithm;

*e*_*i*_ = associated random error.

### Selection criteria for models

Biomass models were evaluated by five criteria widely used on assessing goodness of fit in regression models, which were also used for DM predictions analysis (Table 2). All of them consider in their formulas the difference between actual value and the respective estimate, i.e., the residual or error *e*_*i*_ *.* Therefore, they were used here for selecting the model that gave the best estimation among those tested.

**Table 2:** Criteria for model selection of individual tree biomass estimation in the Atlantic Forest biome, Brazil

	Criterion	Formulation	
1	Adjusted coefficient of determination	${R^2}_{adj.}=1-\frac{\left(n-1\right)}{\left(n-k\right)}\left(1-{R}^2\right)$	(7)
		Where: ${R}^2=1-\frac{{\displaystyle \sum_{i-1}^n{e_i}^2}}{{\displaystyle \sum_{i-1}^n{\left({w}_i-\overline{w}\right)}^2}}$	(8)
2	Standard error of estimate	$Syx=\sqrt{\frac{{\displaystyle \sum_{i-1}^n{e_i}^2}}{n-k}}$	(9)
3	Akaike Information Criterion (Akaike [43]) or	$AIC=-2\left(\frac{-n}{2}l\mathrm{n}\left(\frac{1}{n}\sum_{i-1}^n{e_i}^2\right)\right)+2k$	(10)
	Akaike Information Criterion unbiased for small samples^a^, used when $\frac{n}{k}<40$	$AI{C}_c=-2\left(\frac{-n}{2}l\mathrm{n}\left(\frac{1}{n}\sum_{i-1}^n{e_i}^2\right)\right)+2k\frac{n}{\left(n-k-1\right)}$	(11)
4	Information Criterion or Bayesian Schwartz (Schwartz, [44])	$BIC=-2\left(\frac{-n}{2}l\mathrm{n}\left(\frac{1}{n}\sum_{i-1}^n{e_i}^2\right)\right)+l\mathrm{n}(n)k$	(12)
5	Residual Analysis	*r*_*i*_ = (*w*_*i*_ − *ŵ*_*i*_)	(13)

^a^According to Burnham and Anderson [45]. Where: *n* = number of cases; *k* = number of parameters of the model

*ŵ*_*i*_ = Estimated biomass. *w*_*i*_ = Actual biomass; $\overline{w}$ = average observed biomass

In *AIC*, *AIC*_*c*_ and *BIC k* must be increased by 1, which refers to a degree of freedom for the variance
